# Understanding the bidirectional association between obesity and risk of psychological distress and depression in young adults in the US: available evidence, knowledge gaps, and future directions

**DOI:** 10.3389/fpsyt.2024.1422877

**Published:** 2025-01-10

**Authors:** Michael Friedman, Ryan Chang, Zahir Malik Amin, Tanuja Rajan, Rahul Singh, Samuel Yousefzai, Izza Shahid, Khurram Nasir, Zulqarnain Javed

**Affiliations:** ^1^ George Washington University School of Medicine, Washington, DC, United States; ^2^ Baylor College of Medicine, Houston, TX, United States; ^3^ School of Medicine, University of Texas Medical Branch, Galveston, TX, United States; ^4^ Department of Internal Medicine, University of North Carolina Health Southeastern, Lumberton, NC, United States; ^5^ Cardiovascular Division, University of Minnesota, Minneapolis, MN, United States; ^6^ University of Notre Dame, Notre Dame, IN, United States; ^7^ Center for Cardiovascular Computation and Precision Health, DeBakey Heart and Vascular Center, Houston, TX, United States

**Keywords:** obesity, depression, psychological distress (PD), young adults, cardiovascular health, social determinants of health (SDOH), mental health

## Abstract

While the physical health effects of obesity are well-characterized, an emerging branch of research has shown that obesity additionally plays a critical role in one’s mental health. Young adults, in a pivotal transition phase in their lives, may be particularly prone to the concurrent effects of obesity and adverse mental health outcomes. The purpose of this review is to comprehensively examine existing data regarding the connection between obesity and two widely validated measures of mental health: psychological distress and depression. The connection between mental health outcomes and obesity is mediated by a complex interplay between biological and sociocultural factors, which is explored in this review with particular focus on younger adults aged 20-39. Further, the impact of several demographic factors including race/ethnicity, gender, and immigration status are examined closely. To our knowledge, this review is one of the first efforts to integrate existing knowledge between obesity and mental health, with particular regard for young adults and the impact of other key sociodemographic characteristics. This review has important implications at the interface of two of the most pressing public health crises in the United States.

## 1 Introduction

Obesity is an emerging public health crisis in the United States, and the rates of obesity have continued to rise substantially in recent decades. Obesity in adults aged 20 and over increased from 30.5% to 42.4% between 1999-2000 and 2017-2018, with the prevalence of severe obesity (BMI > 40.0) nearly doubling in this same time period (1). While the physical health risks associated with obesity—including cardiovascular disease (CVD), type 2 diabetes mellitus (T2DM), and cancer—are well characterized, the mental health effects of obesity remain less understood (2, 3).

Several studies have established that obesity is strongly associated with adverse mental health outcomes, including major depressive disorder (MDD) (4–9). Adolescents and young adults (AYAs), at a pivotal stage in their physical and neurological development, experience an increased risk of these mental health effects, as well as the concurrent effects of obesity. According to the CDC, 45.3% of adults aged 20-39 with depression also experience obesity, while just 30.0% of non-depressed individuals within this age group are obese (7).

The purpose of this review is to summarize emerging evidence regarding the intricate bidirectional association in AYAs between obesity and two widely validated measures of mental health: psychological distress (PD) and depression. To our knowledge, this review is one of the first efforts to integrate existing knowledge between obesity and mental health, with particular regard for younger populations and the impact of other key sociodemographic characteristics.

## 2 Depression, psychological distress, and obesity: background and current trends

### 2.1 Measurement of depression and psychological distress in adolescents and young adults

This review focuses on studies that have evaluated major depressive disorder (MDD) and psychological distress (PD) according to the following criteria:

MDD is formally defined by the Diagnostic and Statistical Manual of Mental Disorders, Fifth Edition (DSM-5) criteria. According to the DSM-5, MDD is characterized by the presence of five or more characteristic depressive symptoms during the same 2-week period, representing a change from previous functioning, with at least one of the symptoms being either depressed mood or anhedonia (loss of interest or pleasure) (10).

PD encompasses symptoms of anxiety, depression, and other emotional suffering, which can significantly impact a person’s daily functioning and quality of life. Early evaluation and screening for distress are crucial for timely management and improved medical outcomes, which is typically performed through the Kessler Psychological Distress Scale (11, 12).

### 2.2 Classification of obesity

According to the CDC, an individual is classified as obese if their body mass index (BMI) is ≥ 30 kg/m^2^. Obesity is further divided into subcategories including Class 1 (BMI 30-34.99 kg/m^2^), Class 2 (BMI 35-39.99 kg/m^2^), and Class 3 (BMI >40 kg/m^2^).

### 2.3 Current evidence of PD/MDD in AYA population with obesity

Several population-based studies have established an association between mental health and obesity. In 2010, a meta-analysis of over 58,000 participants established a bi-directional association between depression and obesity that was statistically significant among adults aged 20-59 (8). The mixed aggregation of adults across all age categories limited the ability to make specific conclusions about younger adults, which was a consistent limitation seen across many of the studies that have been published in this area to date. Interestingly, the obesity/MDD association was not found to be statistically significant in individuals <20 years, further emphasizing the importance of age-related considerations in this area of research. An additional meta-analysis from De Wit et al. (13) and a cross-sectional study by Ul-Haq et al. again established a significant association between MDD and obesity – especially among females – but the age-related effects of this relationship were still unclear. Some studies indicate that this relationship may be especially potent in younger populations, though more of these studies have focused on adolescents rather than the impact on young adult populations (9, 14, 15).

**Table 1** summarizes existing research in this area and the limitations of current data. Our findings point to a need for continued age-related considerations in this area of research for several reasons. First, studies indicate several key distinctions in the PD/MDD-obesity relationship across age groups. AYA populations are typically not as physically active and experience increased levels of psychosocial stress compared to pre-adolescents (16, 17). Weight changes and differences in appetite are more common in adolescent MDD than adult MDD (18), contributing to elevated risk of obesity among depressed AYAs. Other studies have indicated that early childhood traumatic events contribute to elevated risk of CVD and obesity in adulthood (14, 15, 19). There are additional neurological differences between AYA and adult depression, including differences in cortisol levels. While hypercortisolemia is commonly identified in adult patients with MDD, this same finding does not hold in AYA’s with MDD (20). Incidence of concurrent obesity and depression further varies depending on the subtype of depression. Individuals with atypical MDD– a form of depression characterized by mood improvements following positive experiences– are more likely to experience obesity than those with typical MDD (21). These findings indicate that the mental health/obesity association may be particularly potent in AYA populations, signifying a need for more age-specific considerations in this area of research.

**Table 1:** Key population based studies establishing the obesity-MDD association.

Study	Methods, Population Sample	Key Findings	Potential Limitations
Luppino et al., 2010 (8)	Meta analysis of 15 studies, including a total of 58,475 participants	• Baseline obesity increased risk of depression in follow-up screening (95% CI, 1.22-1.98) • A relationship was still seen, though less severe among overweight individuals and depressive risk (95% CI, 1.07- 1.51). • While this association held across adults aged 20-59, the same relationship was not found in individuals <20 years old.	• The study was unable to make any conclusions regarding covariates other than age and sex.
Kubzansky et al., 2012 (9)	Prospective study of 1,528 adolescents in Cincinnati. Psychological distress and BMI were measured annually for 4 years.	• The severely obese group demonstrated the highest levels of anxiety and depressive symptoms. • Higher psychological distress levels were seen in participants with higher baseline BMI. • There was little change in BMI over the course of the study, regardless of baseline depression/anxiety status.	• 95% of the participants were either non-Hispanic black or white, limiting the generalizability of these findings across ethnic groups.
Ul-Haq et al., 2014 (16)	Cross-sectional study of 140,564 participants from UK Biobank who were assessed for mood disorder.	• Women with higher BMI experienced higher risk of depression, with further risk elevation depending on obesity subclass. • While women experienced increased risk of obesity regardless of subclass, only Class III obese men exhibited significantly increased risk of depression.	• This study was exclusively on adults aged 40- 69 years, limiting its generalizability to younger populations
De Wit et al., 2010 (17)	Meta-analysis of cross- sectional studies, totaling out to 204,507 participants to determine potential association between depression and obesity.	• There is a significant positive relationship between obesity and depression, with a more marked relationship among women • Other subgroup mediators including age did not affect this association.	• The work does not consider variation by race/ethnicity. • The analysis does not address the potential underlying causal mechanism of this relationship.
Polanka et al., 2017 (18)	17,787 adults who were non-obese were examined over 3 years to assess MDD and obesity outcomes.	• Atypical MDD (OR 1.68) and dysthymic disorder (OR 1.66) were more strongly associated with obesity than typical MDD. • Hispanic and Latino individuals experienced the strongest atypical MDD and obesity relationship.	• Study relies on self-reported height and weight recordings.

## 3 Current biological and psychological hypotheses for obesity/MDD relationship

### 3.1 Systemic inflammation hypotheses

Numerous studies have indicated that nutritious diets reduce inflammatory processes in the brain (22–24). Calorie-rich diets with high levels of saturated fats and sugar have been shown to stimulate immune activation and negatively affect hippocampal function and other cognitive processes (25, 26). Diets high in refined carbohydrates and saturated fat have additionally been shown to reduce brain-derived neurotrophic factor (BDNF) in the hippocampus, resulting in impaired spatial memory and increased risk of depression (22, 27, 28). Adipocytes initiate the release of pro-inflammatory cytokines including interleukin-6, interleukin-2, and C-reactive protein, which have an established role in inducing psychological distress (29). Longitudinal studies have further indicated that individuals scoring lower on their Dietary Inflammatory Index experience lower incidence of depression (30), and that anti-inflammatory drugs may alleviate some depressive symptoms (31, 32). These findings point to a marked relationship between diet quality, chronic inflammation, and concurrent obesity/mental health outcomes.

### 3.2 HPA axis hypothesis

The association between obesity and depression appears to involve hypothalamic-pituitary-adrenal (HPA) axis dysregulation. Individuals with MDD commonly experience chronically elevated levels of cortisol, increasing the likelihood of HPA axis dysfunction (33, 34). Overstimulation of the HPA axis ultimately causes glucocorticoid receptors to become less sensitive (29), and subsequent inadequate negative feedback mechanisms result in continued production of stress hormones that contribute to increased obesity risk (29). The HPA-axis hypothesis may be especially critical in younger populations, as past research has established that cortisol levels vary depending on age and correspondingly may influence depression susceptibility (23, 35).

### 3.3 Self-medication hypothesis and maladaptive coping

A “self-medication” hypothesis may also play a role in connecting MDD and obesity (19, 22). According to this theory, unhealthy foods provide temporary relief from stress, increasing the likelihood of “subtle addiction” (19). This can result in poor eating habits and increase the likelihood of concurrent depression and obesity, as revealed by the systemic inflammation hypothesis (See section 3.2) (22). Obese AYAs additionally experience increased risk of substance abuse as a coping mechanism for negative emotions (36).

Psychosocial stressors may contribute to additional maladaptive coping mechanisms, including suboptimal sleep patterns (37–39). Poor sleep quality has been linked to both increased risk of depression and obesity (40), and these stress-related disruptions to sleeping patterns seemingly play an especially important role in the mental well-being of younger populations (41–45).

## 4 Variation by sociodemographic factors

### 4.1 Variation by gender

Across all age groups, both PD and MDD are more common in females than males (46–48). This disparity is believed to be most pronounced during adolescence (49), and approximately one in six females aged 12-20 have experienced at least one episode of MDD (50). While obesity rates among males and females are similar between different age groups (1), females are more likely to experience effects of obesity-related diseases (16). Females may further experience higher levels of weight-related stigma compared to males, increasing the likelihood of adverse mental health outcomes (47). As such, females additionally are more likely to resort to extreme dieting practices that negatively impact bodily health and psychological well-being (47, 51). Adolescent females are also more likely to resort to negative coping mechanisms for weight concerns, including smoking (52).

Across all age groups, females with MDD are more likely to experience obesity than females without depression, yet this trend does not hold in males (7). A study of adults with MDD found that depression in females was positively linked to BMI, total body fat, and visceral fat, but these associations were not found to be statistically significant in males (53). Additionally, adolescent obese females have displayed a nearly four-times higher risk of MDD (HR=3.9) compared to normal weight individuals, significantly stronger than the association seen in males (HR=1.5) (54, 55).

Males have been found to be less likely to report weight dissatisfaction than females (56), though male experiences of stigma also contribute to substantially increased likelihood of psychological distress and depression (57, 58). Experiencing weight-related stigma is more common in younger populations, irrespective of gender (49, 57, 59). These experiences of stigmatization contribute to elevated risk of PD and lower levels of self-esteem in young populations (59).

### 4.2 Variation by race/ethnicity

The burden of obesity is increasing in younger populations across all racial groups in the U.S., but non-Hispanic Black (NHB) and Hispanic American adults experience particularly high rates of obesity (60).

Evidence additionally suggests that NHB and Hispanic individuals tend to have higher levels of PD than non-Hispanic White (NHW) individuals, as well as higher rates of MDD (61, 62). Studies indicate that variation in socioeconomic status (SES), psychosocial stressors, limited access to healthcare resources, and experiences of racial discrimination may contribute to increased likelihood of concurrent MDD/PD and obesity among minority groups (62–64). In addition, barriers to treatment and diagnosis—including structural factors, lack of accessibility, and stigmatization—may contribute to significant underdiagnosis among racial/ethnic minorities (61, 63, 65).

More research is needed to identify racial/ethnic disparities in the progression of the MDD/obesity relationship. While these studies establish the current racial disparities in both obesity and mental health outcomes, there is limited evidence regarding the specific association between obesity and mental health outcomes in young adults. Future research should focus in particular on young adult minority populations, where current evidence is scarce.

### 4.3 Variation by immigration/citizenship status

The association between MDD and obesity varies depending on both immigration status and length of U.S. residency. Studies have further indicated that the MDD/obesity relationship in immigrant populations may differ from that seen in other socio-demographic groups (38). Surprisingly, a study of Brazilian, Haitian, and Latina immigrants found that females with higher levels of occupational physical activity had a higher likelihood of depression, despite experiencing lower rates of obesity (66). This result may suggest that other variables contribute to higher levels of depression among immigrants in physically active workplaces, such as physical strain, workplace demand, or employer discrimination (66).

Other studies have described an “immigrant paradox,” in which immigrant youth are less likely to experience mental health complications than US-born youth despite the elevated psychosocial risk factors associated with immigration (67, 68). However, this paradox does not appear to hold for obesity, as studies have indicated that children of immigrant mothers are more likely to be obese than the offspring of US-born mothers (69). These results, however, may vary by generational status of the immigrant population. In particular, obesity is less common in first-generation immigrant adolescents than second- or third-generation adolescents (70), indicating that more acculturated immigrants may be at higher risk of becoming obese. Additional studies have determined that relative obesity risk may be associated with the length of residency in the U.S (71–73).

## 5 Social determinants of health - potential upstream drivers of obesity, psychological distress, and depression

**Figure 1:**
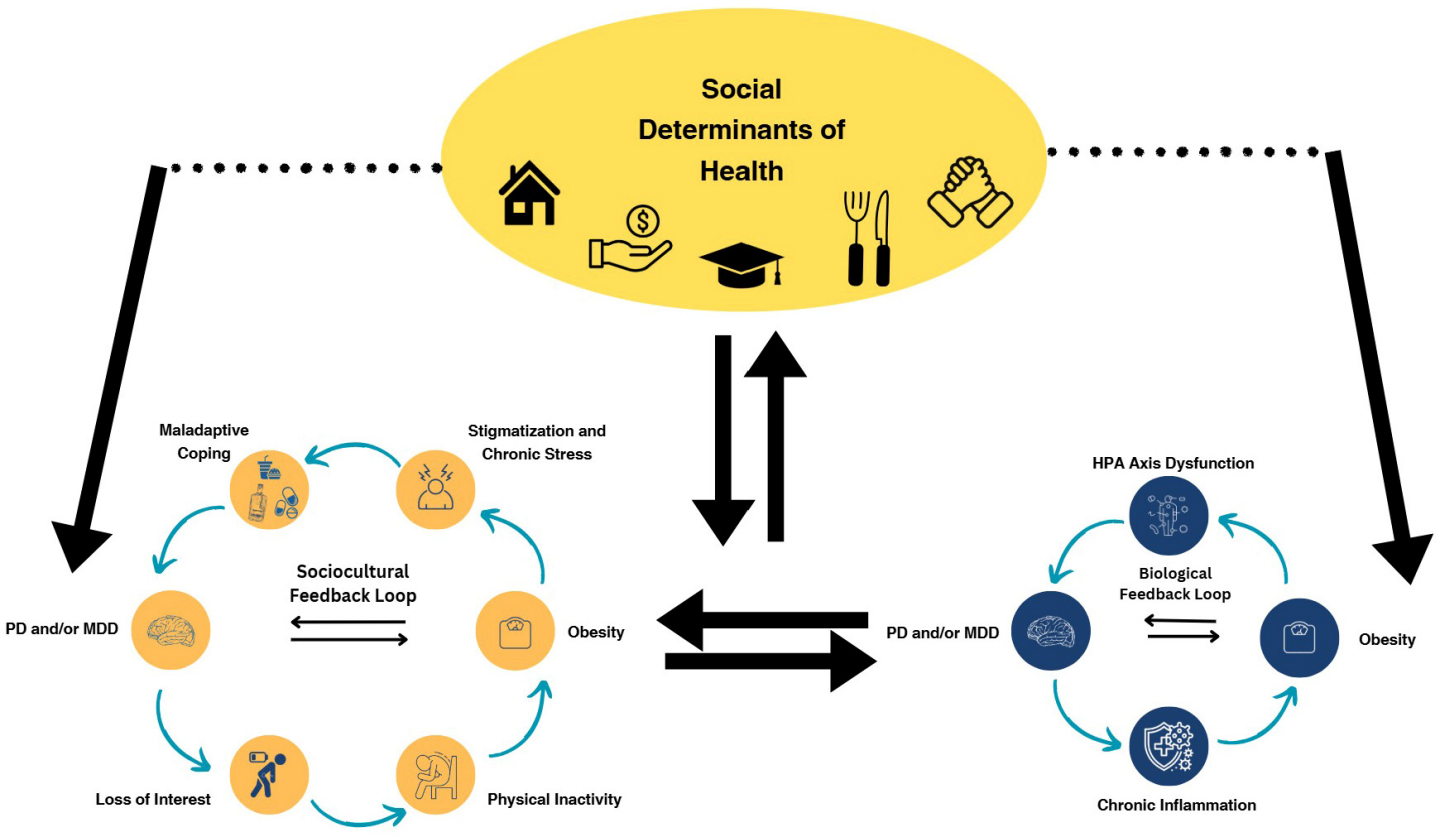
The PD/obesity association is upheld by several feedback loops, creating a vicious cycle where the two conditions reinforce one another. At play are both a sociocultural feedback loop involving several key dynamics including stigmatization, chronic stress, and maladaptive coping mechanisms such as substance abuse and binge eating. The biological association between PD and obesity is characterized by HPA axis dysfunction, excessive cortisol release, and systemic inflammation. Both of these feedback loops may be initiated by broader social determinants of health such as food insecurity, social support, and socioeconomic status.

As shown in **Figure 1**, the association between obesity and mental health outcomes is mediated by a complex interplay between biological and sociocultural factors, as well as upstream social determinants of health (SDOH). Evidence suggests that SDOH such as socioeconomic status, food insecurity, experiences of discrimination, social support, and housing conditions are strongly associated with both physical and mental health outcomes. Over 15% of individuals living in poverty also experience depression, compared to just 3.5% for those over 400% above the poverty line (74). Studies have proposed a “social mobility hypothesis” in which increased SES mitigates the mental health risk that one faces while in a lower SES bracket (75). According to this hypothesis, younger populations are at higher risk of mental health disorders due to less mobility in their SES (75), emphasizing the importance of age-specific considerations when analyzing depression risk.

Additional SDOH associated with depression and obesity include social support, housing conditions, food insecurity, and experiences of discrimination (76, 77). Studies have indicated that there is a three-fold increase in depression incidence among individuals who reported being distant from family members (77), as well as a graded increase in obesity risk by relative SDOH burden (78). Strikingly, 12.8% of U.S. households still reported being food insecure at times in 2022 (79), which is strongly associated with obesity risk, malnutrition, and higher SDOH burden (80–82). Experiences of childhood food insecurity have additionally been shown to increase the likelihood of both obesity and adverse mental health outcomes later in life (83).

To date, there is still limited evidence regarding the specific impacts of these SDOH on AYAs. Future research should more thoroughly establish how the impacts of these SDOH vary depending on age group, and how they differentially contribute to obesity and mental health outcomes.

## 6 Discussion and future directions

The change from adolescence to adulthood is a dynamic and complex developmental stage, during which the relationship between PD/MDD-obesity must be studied in detail. Our research establishes that this stage of life is a period of unique vulnerability to the concurrent effects of obesity and mental health outcomes, and that this association appears to be further exacerbated by key demographic characteristics including racial/ethnic, gender, and immigration status. While the association between MDD and obesity is well-characterized, our work indicates that the AYA population is often not well-represented in this area of research.

To effectively address the public health crises of obesity and mental illness, policies should emphasize the intertwined nature of these conditions, and the additional heightened vulnerability of AYAs to these effects. Health systems should partner with community stakeholders to confront challenges faced by AYAs with obesity, especially those from underserved backgrounds, and design evidence-based interventions to enhance access to preventive health services while addressing social and structural barriers in obesity and psychiatric care. These include obesity and psychiatric illness screening, nutritional counseling, social support, transportation, access to medical and psychological therapies, addressing cost barriers, and implementing strategies to address medication non-adherence in the AYA population.

## 7 Conclusions

Among AYAs, MDD and PD have an intricate interplay with obesity which may be distinct from their mutual association in the adult population. To date, research specifically examining this association in AYA populations is limited, pointing to a need for more research specifically considering the obesity/mental health association in young populations. SDOH such as housing, food insecurity, socioeconomic status, and discrimination have been shown to increase the risk of MDD and PD, although their potency in AYA is less clear. Factors involved in this include a dysregulated HPA axis, systemic inflammation, and the “self-medication” hypothesis. There is further nuanced variation based on gender, race/ethnicity and immigration/citizenship status, but age-related association remains somewhat unclear. These findings have important implications at the interface of two of the most pressing emerging public health crises in the U.S.
